# A Bayesian reanalysis of the Standard versus Accelerated Initiation of Renal-Replacement Therapy in Acute Kidney Injury (STARRT-AKI) trial

**DOI:** 10.1186/s13054-022-04120-y

**Published:** 2022-08-25

**Authors:** Fernando G. Zampieri, Bruno R. da Costa, Suvi T. Vaara, François Lamontagne, Bram Rochwerg, Alistair D. Nichol, Shay McGuinness, Danny F. McAuley, Marlies Ostermann, Ron Wald, Sean M. Bagshaw, STARRT-AKI Investigators, Sean M. Bagshaw, Ron Wald, Neill K. J. Adhikari, Rinaldo Bellomo, Didier Dreyfuss, Bin Du, Martin P. Gallagher, Stéphane Gaudry, François Lamontagne, Michael Joannidis, Kathleen D. Liu, Daniel F. McAuley, Shay P. McGuinness, Alistair D. Nichol, Marlies Ostermann, Paul M. Palevsky, Haibo Qiu, Ville Pettilä, Antoine G. Schneider, Orla M. Smith, Suvi Vaara, Matthew Weir, Rinaldo Bellomo, Glenn M. Eastwood, Leah Peck, Helen Young, Peter Kruger, Gordon Laurie, Emma Saylor, Jason Meyer, Ellen Venz, Krista Wetzig, Craig French, Forbes McGain, John Mulder, Gerard Fennessy, Sathyajith Koottayi, Samantha Bates, Miriam Towns, Rebecca Morgan, Anna Tippett, Andrew Udy, Chris Mason, Elisa Licari, Dashiell Gantner, Jason McClure, Alistair Nichol, Phoebe McCracken, Jasmin Board, Emma Martin, Shirley Vallance, Meredith Young, Chelsey Vladic, Steve McGloughlin, David Gattas, Heidi Buhr, Jennifer Coles, Debra Hutch, James Wun, Louise Cole, Christina Whitehead, Julie Lowrey, Kristy Masters, Rebecca Gresham, Victoria Campbell, David Gutierrez, Jane Brailsford, Loretta Forbes, Lauren Murray, Teena Maguire, Martina NiChonghaile, Neil Orford, Allison Bone, Tania Elderkin, Tania Salerno, Tim Chimunda, Jason Fletcher, Emma Broadfield, Sanjay Porwal, Cameron Knott, Catherine Boschert, Julie Smith, Angus Richardson, Dianne Hill, Graeme Duke, Peter Oziemski, Santiago Cegarra, Peter Chan, Deborah Welsh, Stephanie Hunter, Owen Roodenburg, John Dyett, Nicos Kokotsis, Max Moser, Yang Yang, Laven Padayachee, Joseph Vetro, Himangsu Gangopadhyay, Melissa Kaufman, Angaj Ghosh, Simone Said, Alpesh Patel, Shailesh Bihari, Elisha Matheson, Xia Jin, Tapaswi Shrestha, Kate Schwartz, Martin P. Gallagher, Rosalba Cross, Winston Cheung, Helen Wong, Mark Kol, Asim Shah, Amanda Y. Wang, Zoltan Endre, Celia Bradford, Pierre Janin, Simon Finfer, Naomi Diel, Jonathan Gatward, Naomi Hammond, Anthony Delaney, Frances Bass, Elizabeth Yarad, Hergen Buscher, Claire Reynolds, Nerilee Baker, Michael Joannidis, Romuald Bellmann, Andreas Peer, Julia Hasslacher, Paul Koglberger, Sebastian Klein, Klemens Zotter, Anna Brandtner, Armin Finkenstedt, Adelheid Ditlbacher, Frank Hartig, Dietmar Fries, Mirjam Bachler, Bettina Schenk, Martin Wagner, Philipp Eller, Thomas Staudinger, Esther Tiller, Peter Schellongowski, Andja Bojic, Eric A. Hoste, Stephanie Bracke, Luc De Crop, Daisy Vermeiren, Fernando Thome, Bianca Chiella, Lucia Fendt, Veronica Antunes, Jean-Philippe Lafrance, François Lamontagne, Frédérick D’Aragon, Charles St-Arnaud, Michael Mayette, Élaine Carbonnaeu, Joannie Marchand, Marie-Hélène Masse, Marilène Ladouceur, Alexis F. Turgeon, François Lauzier, David Bellemare, Charles Langis Francoeur, Guillaume LeBlanc, Gabrielle Guilbault, Stéphanie Grenier, Eve Cloutier, Annick Boivin, Charles Delisle-Thibault, Panagiota Giannakouros, Olivier Costerousse, Jean-François Cailhier, François-Martin Carrier, Ali Ghamraoui, Martine Lebrasseur, Fatna Benettaib, Maya Salamé, Dounia Boumahni, Ying Tung Sia, Jean-François Naud, Isabelle Roy, Henry T. Stelfox, Stacey Ruddell, Braden J. Manns, Shelley Duggan, Dominic Carney, Jennifer Barchard, Richard P. Whitlock, Emilie Belley-Cote, Nevena Savija, Alexandra Sabev, Troy Campbell, Thais Creary, Kelson Devereaux, Shira Brodutch, Claudio Rigatto, Bojan Paunovic, Owen Mooney, Anna Glybina, Oksana Harasemiw, Michelle Di Nella, John Harmon, Navdeep Mehta, Louis Lakatos, Nicole Haslam, Francois Lellouche, Mathieu Simon, Ying Tung, Patricia Lizotte, Pierre-Alexandre Bourchard, Bram Rochwerg, Tim Karachi, Tina Millen, John Muscedere, David Maslove, J. Gordon Boyd, Stephanie Sibley, John Drover, Miranda Hunt, Ilinca Georgescu, Randy Wax, Ilan Lenga, Kavita Sridhar, Andrew Steele, Kelly Fusco, Taneera Ghate, Michael Tolibas, Holly Robinson, Matthew A. Weir, Ravi Taneja, Ian M. Ball, Amit Garg, Eileen Campbell, Athena Ovsenek, Sean M. Bagshaw, Sean van Diepen, Nadia Baig, Sheldon Magder, Han Yao, Ahsan Alam, Josie Campisi, Erika MacIntyre, Ella Rokosh, Kimberly Scherr, Stephen Lapinsky, Sangeeta Mehta, Sumesh Shah, Daniel J. Niven, Henry T. Stelfox, Stacey Ruddell, Michael Russell, Kym Jim, Gillian Brown, Kerry Oxtoby, Adam Hall, Luc Benoit, Colleen Sokolowski, Bhanu Prasad, Jag Rao, Shelley Giebel, Demetrios J. Kutsogiannis, Patricia Thompson, Tayne Thompson, Robert Cirone, Kanthi Kavikondala, Mark Soth, France Clarke, Alyson Takaoka, Ron Wald, David Mazer, Karen Burns, Jan Friedrich, David Klein, Gyan Sandhu, Marlene Santos, Imrana Khalid, Jennifer Hodder, Peter Dodek, Najib Ayas, Victoria Alcuaz, Gabriel Suen, Oleksa Rewa, Gurmeet Singh, Sean Norris, Neil Gibson, Castro Arias, Aysha Shami, Celine Pelletier, Neill K. J. Adhikari, Alireza Zahirieh, Andre Amaral, Nicole Marinoff, Navjot Kaur, Adic Perez, Jane Wang, Gregory Haljan, Christopher Condin, Lauralyn McIntyre, Brigette Gomes, Rebecca Porteous, Irene Watpool, Swapnil Hiremath, Edward Clark, Margaret S. Herridge, Felicity Backhouse, M. Elizabeth Wilcox, Karolina Walczak, Vincent Ki, Asheer Sharman, Martin Romano, Sean M. Bagshaw, R. T. Noel Gibney, Adam S. Romanovsky, Oleksa Rewa, Lorena McCoshen, Nadia Baig, Gordon Wood, Daniel Ovakim, Fiona Auld, Gayle Carney, Meili Duan, Xiaojun Ji, Dongchen Guo, Zhili Qi, Jin Lin, Meng Zhang, Lei Dong, Jingfeng Liu, Pei Liu, Deyuan Zhi, Guoqiang Bai, Yu Qiu, Ziqi Yang, Jing Bai, Zhuang Liu, Haizhou Zhuang, Haiman Wang, Jian Li, Mengya Zhao, Xiao Zhou, Xianqing Shi, Baning Ye, Manli Liu, Jing Wu, Yongjian Fu, Dali Long, Yu Pan, Jinlong Wang, Huaxian Mei, Songsong Zhang, Mingxiang Wen, Enyu Yang, Sijie Mu, Jianquan Li, Tingting Hu, Bingyu Qin, Min Li, Cunzhen Wang, Xin Dong, Kaiwu Wang, Haibo Wang, Jianxu Yang, Bin Du, Chuanyao Wang, Dongxin Wang, Nan Li, Zhui Yu, Song Xu, Lan Yao, Guo Hou, Zhou Liu, Liping Lu, Yingtao Lian, Chunting Wang, Jichen Zhang, Ruiqi Ding, Guoqing Qi, Qizhi Wang, Peng Wang, Zhaoli Meng, Man Chen, Xiaobo Hu, Xiandi He, Shibing Zhao, Lele Hang, Rui Li, Suhui Qin, Kun Lu, Shijuan Dun, Cheng Liu, Qi Zhou, Zhenzhen Chen, Jing Mei, Minwei Zhang, Hao Xu, Jincan Lin, Qindong Shi, Lijuan Fu, Qinjing Zeng, Hongye Ma, Jinqi Yan, Lan Gao, Hongjuan Liu, Lei Zhang, Hao Li, Xiaona He, Jingqun Fan, Litao Guo, Yu Liu, Xue Wang, Jingjing Sun, Zhongmin Liu, Juan Yang, Lili Ding, Lulu Sheng, Xingang Liu, Jie Yan, Quihui Wang, Yifeng Wang, Dan Zhao, Shuangping Zhao, Chenghuan Hu, Jing Li, Fuxing Deng, Haibo Qui, Yi Yang, Min Mo, Chun Pan, Changde Wu, Yingzi Huang, Lili Huang, Airan Liu, Ville Pettilä, Suvi T. Vaara, Anna-Maija Korhonen, Sanna Törnblom, Sari Sutinen, Leena Pettilä, Jonna Heinonen, Eliria Lappi, Taria Suhonen, Sari Karlsson, Sanna Hoppu, Ville Jalkanen, Anne Kuitunen, Markus Levoranta, Jaakko Långsjö, Sanna Ristimäki, Kaisa Malila, Anna Wootten, Simo Varila, Mikko J. Järvisalo, Outi Inkinen, Satu Kentala, Keijo Leivo, Paivi Haltia, Didier Dreyfuss, Jean-Damien Ricard, Jonathan Messika, Abirami Tiagarajah, Malo Emery, Aline Dechanet, Coralie Gernez, Damien Roux, Laurent Martin-Lefevre, Maud Fiancette, Isabelle Vinatier, Jean Claude Lacherade, Gwenhaël Colin, Christine Lebert, Marie-Ange Azais, Aihem Yehia, Caroline Pouplet, Matthieu Henry- Lagarrigue, Amélie Seguin, Laura Crosby, Julien Maizel, Dimitri Titeca-Beauport, Alain Combes, Ania Nieszkowska, Paul Masi, Alexandre Demoule, Julien Mayaux, Martin Dres, Elise Morawiec, Maxens Decalvele, Suela Demiri, Morgane Faure, Clémence Marios, Maxime Mallet, Marie Amélie Ordon, Laura Morizot, Marie Cantien, François Pousset, Stéphane Gaudry, Florent Poirson, Yves Cohen, Laurent Argaud, Martin Cour, Laurent Bitker, Marie Simon, Romain Hernu, Thomas Baudry, Sylvie De La Salle, Adrien Robine, Nicholas Sedillot, Xavier Tchenio, Camille Bouisse, Sylvie Roux, Saber Davide Barbar, Rémi Trusson, Fabienne Tamion, Steven Grangé, Dorothée Carpentier, Guillaume Chevrel, Luis Ensenyat-Martin, Sophie Marque, Jean-Pierre Quenot, Pascal Andreu, Auguste Dargent, Audrey Large, Nicolas Chudeau, Mickael Landais, Benoit Derrien, Jean Christophe Callahan, Christophe Guitton, Charlène Le Moal, Alain Robert, Karim Asehnoune, Raphaël Cinotti, Nicolas Grillot, Dominique Demeure, Christophe Vinsonneau, Imen Rahmani, Mehdi Marzouk, Thibault Dekeyser, Caroline Sejourne, Mélanie Verlay, Fabienne Thevenin, Lucie Delecolle, Didier Thevenin, Bertrand Souweine, Elisabeth Coupez, Mireille Adda, Jean-Pierre Eraldi, Antoine Marchalot, Nicolas De Prost, Armand Mekontso Dessap, Keyvan Razazi, Ferhat Meziani, Julie Boisrame-Helms, Raphael Clere-Jehl, Xavier Delabranche, Christine Kummerlen, Hamid Merdji, Alexandra Monnier, Yannick Rabouel, Hassene Rahmani, Hayat Allam, Samir Chenaf, Vincenta Franja, Bertrand Pons, Michel Carles, Frédéric Martino, Régine Richard, Benjamin Zuber, Guillaume Lacave, Karim Lakhal, Bertrand Rozec, Hoa Dang Van, Éric Boulet, Fouad Fadel, Cedric Cleophax, Nicolas Dufour, Caroline Grant, Marie Thuong, Jean Reignier, Emmanuel Canet, Laurent Nicolet, Thierry Boulain, Mai-Anh Nay, Dalila Benzekri, François Barbier, Anne Bretagnol, Toufik Kamel, Armelle Mathonnet, Grégoire Muller, Marie Skarzynski, Julie Rossi, Amandine Pradet, Sandra Dos Santos, Aurore Guery, Lucie Muller, Luis Felix, Julien Bohé, Guillaume Thiéry, Nadia Aissaoui, Damien Vimpere, Morgane Commeureuc, Jean-Luc Diehl, Emmanuel Guerot, Orfeas Liangos, Monika Wittig, Alexander Zarbock, Mira Küllmar, Thomas van Waegeningh, Nadine Rosenow, Alistair D. Nichol, Kathy Brickell, Peter Doran, Patrick T. Murray, Giovanni Landoni, Rosalba Lembo, Alberto Zangrillo, Giacomo Monti, Margherita Tozzi, Matteo Marzaroli, Gaetano Lombardi, Gianluca Paternoster, Michelangelo Vitiello, Shay McGuinness, Rachael Parke, Magdalena Butler, Eileen Gilder, Keri-Anne Cowdrey, Samantha Wallace, Jane Hallion, Melissa Woolett, Philippa Neal, Karina Duffy, Stephanie Long, Colin McArthur, Catherine Simmonds, Yan Chen, Rachael McConnochie, Lynette Newby, David Knight, Seton Henderson, Jan Mehrtens, Stacey Morgan, Anna Morris, Kymbalee Vander Hayden, Tara Burke, Matthew Bailey, Ross Freebairn, Lesley Chadwick, Penelope Park, Christine Rolls, Liz Thomas, Ulrike Buehner, Erin Williams, Jonathan Albrett, Simon Kirkham, Carolyn Jackson, Troy Browne, Jennifer Goodson, David Jackson, James Houghton, Owen Callender, Vicki Higson, Owen Keet, Clive Dominy, Paul Young, Anna Hunt, Harriet Judd, Cassie Lawrence, Shaanti Olatunji, Yvonne Robertson, Charlotte Latimer-Bell, Deborah Hendry, Agnes Mckay-Vucago, Nina Beehre, Eden Lesona, Leanlove Navarra, Chelsea Robinson, Ryan Jang, Andrea Junge, Bridget Lambert, Antoine G. Schneider, Michel Thibault, Philippe Eckert, Sébastien Kissling, Erietta Polychronopoulos, Elettra Poli, Marco Altarelli, Madeleine Schnorf, Samia Abed Mallaird, Claudia Heidegger, Aurelie Perret, Philippe Montillier, Frederic Sangla, Seigenthaller Neils, Aude De Watteville, Mandeep-Kaur Phull, Aparna George, Nauman Hussain, Tatiana Pogreban, Steve Lobaz, Alison Daniels, Mishell Cunningham, Deborah Kerr, Alice Nicholson, Pradeep Shanmugasundaram, Judith Abrams, Katarina Manso, Geraldine Hambrook, Elizabeth McKerrow, Juvy Salva, Stephen Foulkes, Matthew Wise, Matt Morgan, Jenny Brooks, Jade Cole, Tracy Michelle Davies, Helen Hill, Emma Thomas, Marcela Vizcaychipi, Behrad Baharlo, Jaime Carungcong, Patricia Costa, Laura Martins, Ritoo Kapoor, Tracy Hazelton, Angela Moon, Janine Musselwhite, Ben Shelley, Philip McCall, Marlies Ostermann, Gill Arbane, Aneta Bociek, Martina Marotti, Rosario Lim, Sara Campos, Neus Grau Novellas, Armando Cennamo, Andrew Slack, Duncan Wyncoll, Luigi Camporota, Simon Sparkes, Rosalinde Tilley, Austin Rattray, Gayle Moreland, Jane Duffy, Elizabeth McGonigal, Philip Hopkins, Clare Finney, John Smith, Harriet Noble, Hayley Watson, Claire-Louise Harris, Emma Clarey, Eleanor Corcoran, James Beck, Clare Howcroft, Nora Youngs, Elizabeth Wilby, Bethan Ogg, Adam Wolverson, Sandra Lee, Susie Butler, Maryanne Okubanjo, Julia Hindle, Ingeborg Welters, Karen Williams, Emily Johnson, Julie Patrick-Heselton, David Shaw, Victoria Waugh, Richard Stewart, Esther Mwaura, Lynn Wren, Louise Mew, Sara-Beth Sutherland, Jane Adderley, Jim Ruddy, Margaret Harkins, Callum Kaye, Teresa Scott, Wendy Mitchell, Felicity Anderson, Fiona Willox, Vijay Jagannathan, Michele Clark, Sarah Purv, Andrew Sharman, Megan Meredith, Lucy Ryan, Louise Conner, Cecilia Peters, Dan Harvey, Ashraf Roshdy, Amy Collins, Malcolm Sim, Steven Henderson, Nigel Chee, Sally Pitts, Katie Bowman, Maria Dilawershah, Luke Vamplew, Elizabeth Howe, Paula Rogers, Clara Hernandez, Clara Prendergast, Jane Benton, Alex Rosenberg, Lui G. Forni, Alice Grant, Paula Carvelli, Ajay Raithatha, Sarah Bird, Max Richardson, Matthew Needham, Claire Hirst, Jonathan Ball, Susannah Leaver, Luisa Howlett, Carlos Castro Delgado, Sarah Farnell-Ward, Helen Farrah, Geraldine Gray, Gipsy Joseph, Francesca Robinson, Ascanio Tridente, Clare Harrop, Karen Shuker, Derek McLaughlan, Judith Ramsey, Sharon Meehan, Bernd Oliver Rose, Rosie Reece-Anthony, Babita Gurung, Tony Whitehouse, Catherine Snelson, Tonny Veenith, Andy Johnston, Lauren Cooper, Ron Carrera, Karen Ellis, Emma Fellows, Samanth Harkett, Colin Bergin, Elaine Spruce, Liesl Despy, Stephanie Goundry, Natalie Dooley, Tracy Mason, Amy Clark, Gemma Dignam, Geraldine Ward, Ben Attwood, Penny Parsons, Sophie Mason, Michael Margarson, Jenny Lord, Philip McGlone, Luke E. Hodgson, Indra Chadbourn, Raquel Gomez, Jordi Margalef, Rinus Pretorius, Alexandra Hamshere, Joseph Carter, Hazel Cahill, Lia Grainger, Kate Howard, Greg Forshaw, Zoe Guy, Kianoush B. Kashani, Robert C. Albright Jr., Amy Amsbaugh, Anita Stoltenberg, Alexander S. Niven, Matthew Lynch, AnnMarie O’Mara, Syed Naeem, Sairah Sharif, Joyce McKenney Goulart, Matthew Lynch, AnnMarie O’Mara, Syed Naeem, Sairah Sharif, Joyce McKenney Goulart, Ashita Tolwani, Claretha Lyas, Laura Latta, Azra Bihorac, Haleh Hashemighouchani, Philip Efron, Matthew Ruppert, Julie Cupka, Sean Kiley, Joshua Carson, Peggy White, George Omalay, Sherry Brown, Laura Velez, Alina Marceron, Javier A. Neyra, Juan Carlos Aycinena, Madona Elias, Victor M. Ortiz-Soriano, Caroline Hauschild, Robert Dorfman

**Affiliations:** 1 Academic Research Organization, Albert Einstein Hospital, São Paulo, Brazil; 2 Department of Critical Care Medicine, Faculty of Medicine and Dentistry, University of Alberta, 2-124E Clinical Sciences Building, 8440-112 St NW, Edmonton, AB T6G2B7 Canada; 3 Applied Health Research Centre, Li Ka Shing Knowledge Institute of St. Michael’s Hospital, University of Toronto, Toronto, Canada; 4 Division of Intensive Care Medicine, Department of Anesthesiology, Intensive Care and Pain Medicine, University of Helsinki and Helsinki University Hospital, Helsinki, Finland; 5 Department of Medicine, Université de Sherbrooke, Sherbrooke, QC Canada; 6 Centre de Recherche du CHU de Sherbrooke, Sherbrooke, QC Canada; 7 Department of Medicine (Division of Critical Care) and Department of Health Research Methods, Evidence and Impact, McMaster University, Hamilton, ON Canada; 8 Department of Critical Care Medicine, University College Dublin Clinical Research Centre at St. Vincent’s University Hospital, Dublin, Ireland; 9 Monash University, Melbourne, Australia; 10 Cardiothoracic and Vascular Intensive Care Unit, Auckland City Hospital, Auckland and Medical Research Institute of New Zealand, Wellington, New Zealand; 11 The Wellcome-Wolfson Institute for Experimental Medicine, Queen’s University, and The Regional Intensive Care Unit, Royal Victoria Hospital, Belfast, UK; 12 Department of Critical Care Medicine, King’s College London, Guy’s and St Thomas’ Hospital, London, UK; 13 Division of Nephrology, St. Michael’s Hospital, and the Li Ka Shing Knowledge Institute and the University of Toronto, Toronto, Canada

**Keywords:** Bayesian, Kidney-replacement therapy, Acute kidney injury, Mortality, Dialysis, Randomized, Trial

## Abstract

**Background:**

Timing of initiation of kidney-replacement therapy (KRT) in critically ill patients remains controversial. The *Standard versus Accelerated Initiation of Renal-Replacement Therapy in Acute Kidney Injury* (STARRT-AKI) trial compared two strategies of KRT initiation (accelerated versus standard) in critically ill patients with acute kidney injury and found neutral results for 90-day all-cause mortality. Probabilistic exploration of the trial endpoints may enable greater understanding of the trial findings. We aimed to perform a reanalysis using a Bayesian framework.

**Methods:**

We performed a secondary analysis of all 2927 patients randomized in multi-national STARRT-AKI trial, performed at 168 centers in 15 countries. The primary endpoint, 90-day all-cause mortality, was evaluated using hierarchical Bayesian logistic regression. A spectrum of priors includes optimistic, neutral, and pessimistic priors, along with priors informed from earlier clinical trials. Secondary endpoints (KRT-free days and hospital-free days) were assessed using zero–one inflated beta regression.

**Results:**

The posterior probability of benefit comparing an accelerated versus a standard KRT initiation strategy for the primary endpoint suggested no important difference, regardless of the prior used (absolute difference of 0.13% [95% credible interval [CrI] − 3.30%; 3.40%], − 0.39% [95% CrI − 3.46%; 3.00%], and 0.64% [95% CrI − 2.53%; 3.88%] for neutral, optimistic, and pessimistic priors, respectively). There was a very low probability that the effect size was equal or larger than a consensus-defined minimal clinically important difference. Patients allocated to the accelerated strategy had a lower number of KRT-free days (median absolute difference of − 3.55 days [95% CrI − 6.38; − 0.48]), with a probability that the accelerated strategy was associated with more KRT-free days of 0.008. Hospital-free days were similar between strategies, with the accelerated strategy having a median absolute difference of 0.48 more hospital-free days (95% CrI − 1.87; 2.72) compared with the standard strategy and the probability that the accelerated strategy had more hospital-free days was 0.66.

**Conclusions:**

In a Bayesian reanalysis of the STARRT-AKI trial, we found very low probability that an accelerated strategy has clinically important benefits compared with the standard strategy. Patients receiving the accelerated strategy probably have fewer days alive and KRT-free. These findings do not support the adoption of an accelerated strategy of KRT initiation.

**Supplementary Information:**

The online version contains supplementary material available at 10.1186/s13054-022-04120-y.

## Background

Timing of kidney replacement therapy (KRT) initiation in critically ill patients with severe acute kidney injury (AKI) is controversial and has been the focus of several recent randomized trials [1–4]. These trials have been driven by the premise that earlier KRT can facilitate more rapid correction of metabolic, acid–base, and fluid balance derangements, prevent AKI-related complications, and improve clinical outcomes [5–7]. At the same time, KRT is also recognized as an invasive and resource-intensive intervention associated with risks, such as placement of a large central venous catheter, exposure to an extracorporeal circulation, and therapy-related complications, in particular episodes of hemodynamic instability, which may modify the probability of kidney recovery and independence from KRT [2, 3, 8].

The *Standard versus Accelerated Initiation of Renal-Replacement Therapy in Acute Kidney Injury* (STARRT-AKI) trial found no important difference in the primary endpoint of 90-day all-cause mortality when comparing the accelerated with the more conservative strategy for starting KRT in critically ill patients with severe AKI; however, the accelerated strategy conferred greater risk for KRT dependence at 90 days among hospital survivors [3]. The STARRT-AKI trial was designed as a frequentist trial and was interpreted using a traditional framework of null hypothesis testing with a dichotomous interpretation of *p* values under a Neyman–Pearson concept [9]. The reinterpretation of the STARRT-AKI trial through a Bayesian framework may align more naturally with clinician decision-making and provide a more straightforward context, including the provision of direct probabilities of benefit or harm, probabilities of the effect size being within a range of relevant effect sizes, and estimates of equivalence [10–12].

Accordingly, we performed a secondary post hoc analysis of the STARRT-AKI trial data under a Bayesian framework, focusing on assessing the effect of accelerated compared with standard KRT initiation on 90-day all-cause mortality and, secondly, on key kidney-specific outcomes.

## Methods

*Aim, Design and Setting* We performed a post hoc secondary analysis of the STARRT-AKI trial (Data Creation Plan available at: https://www.ualberta.ca/critical-care/research/current-research/starrtaki/documents.html) [3, 13, 14]. In brief, the STARRT-AKI trial randomized critically ill patients greater than 18 years old with kidney dysfunction (serum creatinine level ≥ 1.13 mg per deciliter [100 μmol/l] in women and ≥ 1.47 mg per deciliter [130 μmol/l] in men) and severe AKI to two strategies for KRT initiation. Those allocated to the accelerated strategy were to commence KRT within 12 h of meeting eligibility criteria; the standard strategy entailed deferral of KRT unless a conventional indication for KRT or persistent AKI arose. Details of the protocol, analysis, and findings have been previously reported [3, 13, 14].

*Patients* We included all patients from the modified intention-to-treat analysis (*n* = 2927).

*Endpoints* The primary endpoint was 90-day all-cause mortality. Key secondary endpoints included: (1) number of days alive and free of KRT and (2) days alive and free of hospitalization, both through 90 days. Additional secondary endpoints included: (3) composite for death/KRT at 90 days; (4) KRT dependence at 90 days among survivors; and (5) rehospitalization within 90 days.

*Statistical Analysis* We defined a priori that the model would be a Bayesian Hierarchical model adjusted for the presence of sepsis (Yes/No), type of ICU admission (surgical vs. medical) and baseline chronic kidney disease (CKD) status, defined as premorbid estimated glomerular filtration rate (eGFR) < 60 mL/min/1.73 m^2^ (Yes/No), with study site added as a random intercept [see: data creation plan (DCP) at https://www.ualberta.ca/critical-care/research/current-research/starrtaki/documents.html].

We considered neutral, optimistic, and pessimistic priors. The priors were defined on a log scale for the odds ratio (OR) and assumed a normal distribution. The neutral prior was defined so that 0.95 of the probability mass ranged from an odds ratio between 0.5 and 2.0; that is, it follows a normal distribution defined as *N*(mean, standard deviation) equals to $N\left(0,0.355\right)$. The optimistic and pessimistic priors were mirrored around the effect size that the STARRT-AKI trial was designed to detect (a 6% absolute risk reduction in 90-day all-cause mortality from 40 to 34%, representing an OR = 0.77 [log[OR] =  − 0.257]). Standard deviation was set to consider a 0.15 probability of harm for the optimistic prior and 0.15 probability of benefit for the pessimistic prior; that is, the optimistic prior was centered in a possible benefit (log[OR] = 0.257; OR ~ 0.77), while acknowledging the possibility of harm, and the pessimistic prior was centered at possible harm (log[OR] =  − 0.257; OR ~ 1.30), while considering a 0.15 probability of benefit [9]. Under these assumptions, the optimistic prior was $N\left(-0.257,0.249\right)$ and pessimistic prior was $N\left(0.257,0.249\right)$. Priors for other predictors were set as $N\left(0,1\right)$ for regularization. Default priors for random intercepts in *brms* R package were used [15].

We report the following metrics for the intervention (accelerated strategy) on the primary endpoint: (1) median of the posterior distribution; (2) posterior distribution 95% highest density interval (HDI); (3) probability of direction (PD; the probability that the effect size is on the side of the point estimate); (4) probability of “significance” based on a region of practical equivalence defined using traditional criteria; and (5) probability that the effect size is at least equal to or greater than what was considered as a minimal clinically important difference (MCID) in favor of the intervention, as defined by a survey of the STARRT-AKI international steering committee members (see Additional file 1); (6) probability that the effect size is at least 1.5 times higher than the one defined as MCID (which we considered as a “large” effect). The thresholds beyond which the effect was considered as “significant” were based on a difference in log(OR) that is equivalent of a standardized mean difference of 0.1 in Cohen’s d scale [equivalent to a log(OR) difference of 0.18; to convert from Cohen’s d to standardized log(OR) difference in Cohen’s *d* scale, multiply the log(OR) by $\pi /\sqrt{3}$], which would translate to an odds ratio between 0.83 and 1.19 [16, 17]. These parameters were used to define the region of practical equivalence (ROPE) for this analysis; these values, albeit somewhat arbitrary, are considered as reasonable for equivalence testing [16, 17]. We defined percentage inside ROPE as the proportion of the whole posterior distribution that lies within the ROPE. Convergence and stability of the Bayesian sampling were assessed using R-hat, which should be below 1.01 [13], and effective sample size (ESS), which should be greater than 1000. Models were run using R package *brms* [15] and *emmeans* [18]. All analysis was run in R version 4.2.0.

Further, we also evaluated a secondary set of priors based on observations from earlier trials for the primary outcome, including: (1) the STARRT-AKI pilot trial [19]; (2) the AKIKI and ELAIN trials (given divergent results) [2, 4]; and (3) the individual patient data meta-analysis (IPDMA) (which included all prior trials except the main STARRT-AKI trial) [20].

Secondary endpoints (days alive and KRT-free and days alive and hospital-free) were assessed using a zero–one inflated beta regression models and reported as absolute difference in days between the accelerated and standard strategies (with 95% credible intervals [CrI] of HDI) [21, 22]. We also report the conditional probability of the difference in days alive and KRT-free and hospital-free favoring the accelerated strategy and the probability that the difference is within one day more to one day fewer interval or, secondarily, higher than the consensus MCID. Other secondary binary endpoints were assessed using a similar hierarchical logistic Bayesian model as performed with the primary endpoint. Secondary outcomes were assessed using only neutral priors ($N\left(0,0.355\right)$ for the intervention for the binary component and $N\left(0,1\right)$ for all other variables in the model (see ESM for details), and results are presented as median difference in proportions (with 95% HDI), as well as median OR (with 95% HDI) and the probability of benefit. We report missing values for all outcomes; a complete case analysis was used for all endpoints.

### Consensus for minimal clinically important difference

We surveyed the 24 members of the international steering committee of the STARRT-AKI to generate consensus on a MCID for the primary and secondary endpoints (see Additional file 1). An absolute difference of 0.04 over the baseline event rate of 0.40 for the primary endpoint, all-cause mortality at 90 days, was considered as the MCID (which results in an odds ratio of approximately 0.84; log(OR) =  − 0.175) (see ESM). The margin for a large effect was therefore set as $1.5\times -0.175 \approx 0.26$, which translates to a margin of large effects set as odds ratio below 0.77 or above 1.30. A margin of 3 days was considered as equivalent for the key secondary endpoints.

## Results

### Patients

We studied all 2927 participants (1465 allocated to the accelerated strategy and 1462 to the standard strategy) who were included in the principal modified intention-to-treat analysis presented in the main report of the trial. Mean age was 64.2, and 68% were male. Sepsis was present in 57%, and 77% were receiving mechanical ventilation at the time of randomization. A description of patient characteristics and unadjusted endpoints is shown in Table 1.

**Table 1:** Baseline characteristics, features, and outcomes

Characteristic*	Accelerated, *N* = 1465	Standard, *N* = 1462
Age, mean (SD)	64 (14)	64 (13)
*Sex, n (%)*		
Female	470 (32)	467 (32)
Male	995 (68)	995 (68)
Sepsis, *n* (%)	855 (58)	834 (57)
Surgical admission, *n* (%)	492 (34)	473 (32)
Mechanical ventilation, *n* (%)	1103 (75)	1148 (79)
Creatinine, mean (SD)	121 (92)	118 (87)
Chronic kidney disease, *n* (%)	658 (45)	626 (43)
SOFA score, median (IQR)	12 (9–14)	12 (9–14)
SAPS II score, median (IQR)	57 (45–71)	59 (47–73)
Received KRT, *n* (%)	1418 (97)	903 (62)
Time until KRT, hours, median (IQR)	4 (3–7)	29 (17–68)
Did not receive KRT	48	559
*Outcomes*		
90-day mortality *n* (%)	643 (44)	639 (44)
KRT dependency at 90 days, *n* (%)	85 (10)	49 (6)
KRT dependency or death at 90 days, *n* (%)	728 (50)	688 (47)
Death in hospital, *n* (%)	643 (44)	639 (44)
*Rehospitalization at 90 days, n (%)*		
No	653 (45)	685 (47)
Yes	166 (11)	138 (9)
Days alive and hospital-free at 90 days, medial (IQR)	10 (0, 65)	9 (0, 64)
Days alive and KRT-free at 90 days, median (IQR)	50 (0, 87)	64 (0, 90)

*Missing values not shown in table

### Primary endpoint: all-cause mortality at 90 days

**Fig. 1:**
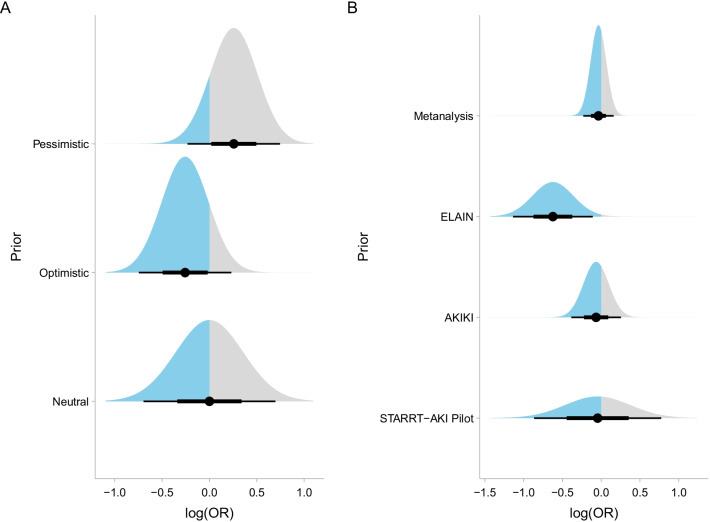
**A** Theoretical priors based on [3] and **B** data-derived priors based on STARRT-AKI pilot, AKIKI, ELAIN and meta-analysis results

**Fig. 2:**
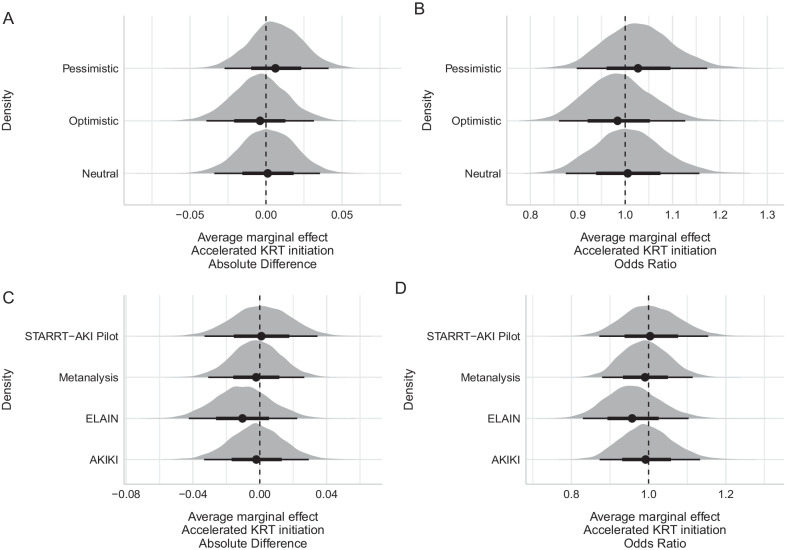
Posterior marginal effects for absolute difference and odds ratio for accelerated strategy. **A** Absolute difference in mortality using theoretical priors. **B** Posterior odds ratio based on theoretical priors. **C** Absolute difference in mortality using data-derived priors. **D** Posterior odds ratio based on data-derived priors. Theoretical priors based on [3] and **B** data-derived priors based on STARRT-AKI pilot, AKIKI, ELAIN, and meta-analysis results

The effect of the intervention on the primary endpoint was minimal, with only minor changes with the use of different priors. The priors used for the primary analysis are graphically shown in Fig. 1, and results for the marginal effects on both absolute and relative (OR) scales are shown in Fig. 2A, B, respectively. The posterior probabilities of effect are shown in Table 2. The results of the full model for the primary outcome using the main sets of priors are shown in Additional file 1: Table S1. There was a high probability that the effect size of the intervention was contained in the region of equivalence defined and a very low (close to zero) probability that the effect of the intervention was large. There was a negligible probability that the intervention was associated with a greater than 0.04 absolute reduction in the primary outcome (consensus MCID). In all scenarios, estimates for the absolute difference were neutral, being 0.13% (95% CrI − 3.30 to 3.40%) for the neutral prior, − 0.39% (95% CrI − 3.46 to 3.00%) for the optimistic prior and 0.64% (95% CrI − 2.53 to 3.88%) for the pessimistic prior, respectively.

**Table 2:** Results for the primary endpoint according to different priors

Prior	Median	HDI 95%	*P* (Benefit)*	%ROPE**	*P* (effect not large)^‡^	*P* (OR < 0.84)^†^	*P* (diff < − 0.04)^⁋^
*Theoretical priors*							
Neutral	1.01	0.87–1.15	0.47	0.99	1.00	0.00	0.01
Optimistic	0.98	0.86–1.12	0.59	0.99	1.00	0.00	0.02
Pessimistic	1.03	0.90–1.18	0.35	0.99	1.00	0.00	0.00
*Data driven priors*							
AKIKI	0.99	0.87–1.13	0.54	0.99	1.00	0.00	0.01
ELAIN	0.96	0.83–1.10	0.73	0.97	1.00	0.00	0.04
Meta-analysis	0.99	0.88–1.12	0.56	0.99	1.00	0.00	0.00
STARRT-AKI Pilot	1.00	0.87–1.15	0.48	0.98	1.00	0.00	0.01

*Probability OR < 1.0. **Probability effect size (OR) is within 0.83–1.19 (equivalence margin). ^‡^Probability effect size is outside a large margin effect of OR between 0.77 and 1.30. ^†^Probability OR is below 0.84 (which results in a 4% reduction in primary outcome). ^⁋^Probability the difference is outcome is greater than 4% favoring accelerated strategy given the data and prior

In the results for the data-derived priors (alternative priors), no scenario provided a posterior probability of benefit above 0.90, and both large effect sizes and effect sizes based on consensus MCID (assessed as both a low OR or a decrease in absolute probability) were very unlikely (Table 2).

### Secondary endpoint: days alive and KRT-free

**Fig. 3:**
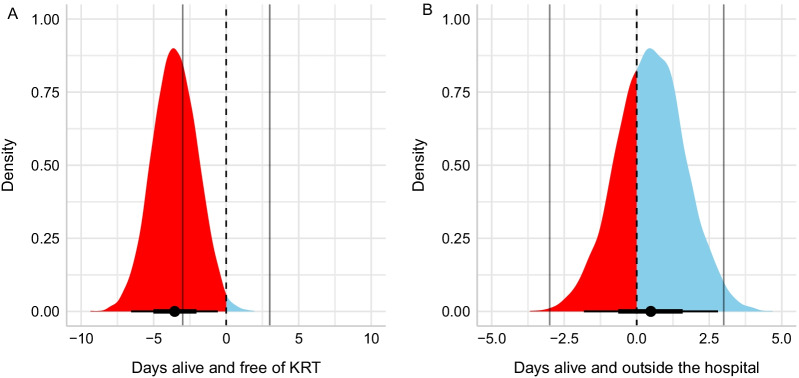
Posterior distribution of **A** days alive and free of KRT and **B** days alive and free of hospitalization. The vertical dashed line represents no difference; values favoring accelerated strategy are shown in blue and probability mass suggesting harm is filled in red. The vertical gray lines at − 3 and + 3 mark what was considered a MCID by the steering committee

The distribution of days alive and KRT-free according to allocated intervention is shown in Additional file 1: Fig. S1, and results for the difference of expected predictions among groups are shown in Fig. 3A. Information was missing for 27 patients (all from the accelerated-strategy group). Patients in the accelerated strategy had fewer days alive and free of KRT, with a median absolute difference of − 3.55 days fewer (95% CrI − 6.38 to − 0.48 days) (Fig. 3A). The probability that the accelerated strategy was associated with more days alive and KRT-free was 0.008, the probability that this difference was within a 1 day fewer to 1 day more range was 0.047, and the probability that this difference was within 3 days fewer to 3 days more range (consensus MCID) was 0.363. The probability that the accelerated strategy was associated with at least 3 more days alive and KRT-free was virtually zero.

### Days alive and hospital-free

The distribution of days alive and free of hospitalization according to allocated intervention is shown in Additional file 1: Fig. S2, and results for the difference of expected predictions among groups are shown in Fig. 3B. Information was missing for 1 patient in the accelerated-strategy group. The accelerated strategy had a median absolute difference of 0.48 days more alive and hospital-free (95% CrI − 1.87; 2.72). The probability that the accelerated strategy was associated with more days alive and hospital-free was 0.657, the probability that this difference was within a 1 day more to 1 day fewer range was 0.566, and the probability that the difference was within a 3 day more to 3 day fewer range (consensus MCID) was 0.983. The probability that the accelerated strategy was associated with at least 3 more days alive and hospital-free was only 0.015.

### Additional secondary endpoints

**Fig. 4:**
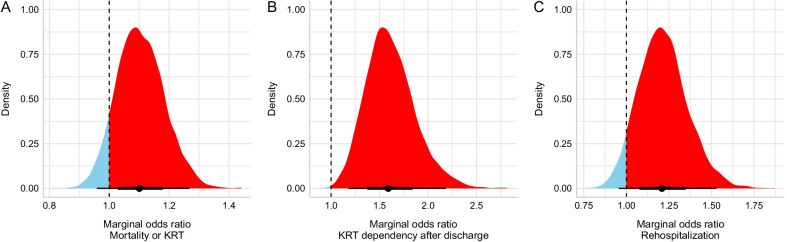
Posterior probability distribution for odds ratio for other secondary endpoints: **A** Mortality or KRT. **B** KRT dependency after discharge. **C** Rehospitalization. The vertical dashed line represents no difference; values favoring accelerated strategy are shown in blue and probability mass suggesting harm is filled in red

The composite endpoint of KRT dependency at 90 days or death was missing in 16 patients (8 in accelerated and 8 in the standard-strategy group). A total of 728 (49.7%) had the composite outcome in the accelerated strategy, and 688 (47.1%) had the composite outcome in standard strategy, respectively (Table 1). The adjusted absolute difference was 2.38% (95% HDI − 1.13 to 5.77%). The median OR was 1.10 (95% HDI 0.95–1.26; Fig. 4A). The posterior probability of benefit with the accelerated strategy was 0.086.

A total of 1,629 patients survived hospital discharge and had KRT data available (814 in the accelerated and 815 in the standard strategy). KRT dependency at 90 days occurred in 85 (10.44%) and 49 (6.01%) patients in the accelerated and standard strategies, respectively, with a median adjusted difference 3.82% (95% HDI 1.40–6.42%) and the median OR was 1.59 (95% HDI 1.15–2.13; Fig. 4B). The posterior probability of benefit with the accelerated strategy was below 0.001.

A total of 1642 patients survived to hospital discharge. Rehospitalization occurred in 166 (20.27%) patients in the accelerated strategy and 138 (16.77%) patients in the standard strategy, respectively. The adjusted difference was 2.87% (95% HDI − 0.50 to 6.57%), and the median OR was 1.21 (95% HDI 0.93–1.49, Fig. 4C). The posterior probability of benefit with the accelerated strategy was 0.056.

## Discussion

In this post hoc Bayesian reanalysis of STARRT-AKI, the largest international randomized trial of acute KRT, we found that the probability that an accelerated strategy was associated with a clinically important or large treatment effect on 90-day all-cause mortality is very low. These findings were consistent across a spectrum of priors used to inform our Bayesian models, including the results from prior trials with conflicting results [1, 2, 4]. In addition, we found high probabilities that the accelerated strategy resulted in fewer KRT-free days, as well as a higher risk of KRT dependence and rehospitalization at 90 days (all probabilities exceeding 0.90) compared with the standard strategy. These findings greatly extend the main frequentist analysis of the STARRT-AKI trial previously reported, by drawing emphasis on the exceedingly low likelihood of any meaningful benefit with a strategy of accelerated KRT initiation [3]. While trials have utilized varying definitions of “accelerated” or “early” and “standard” or “delayed” to define the timing of KRT initiation, the findings of this analysis should strongly reinforce the adoption of a “watch and wait” strategy, where clinician decision-making on when to start KRT for critically ill patients with AKI should be prompted by development of conventional indications, medically refractory complications and/or persistent AKI [3, 21].

The use of Bayesian reanalysis provides a unique opportunity to reappraise, augment, and expand the main results of large, randomized trials using an alternative framework [10]. A Bayesian approach, integrating the concepts of probabilities of benefit or harm for a given intervention, may better mimic how clinicians integrate information to make clinical decisions at the bedside. This may have greater relevance for resource-intensive interventions with known risk profiles, such as KRT [22]. In this reanalysis, we “stressed” the STARRT-AKI trial data with seven different priors for the primary endpoint of 90-day all-cause mortality (with only minor deviations in results). We further provided probabilistic interpretations of the primary and secondary outcomes based not only on thresholds for treatment effect sizes [16, 17], but also by defining a minimal clinically important difference (MCID) from a consensus of the STARRT-AKI trial’s lead investigators.

Establishing a MCID can be challenging. This can often be based on cost-effectiveness analyses or quality-adjusted life years [23] and is increasingly being adopted across disciplines and in clinical trial design [24]. Despite this, there is surprisingly little guidance on how to best define MCID in critical care [25, 26]. We used a very simple consensus analysis based on the expert opinion of the international steering committee of the STARRT-AKI trial [3]. Though imperfect, this approach enabled a global perspective from clinicians who are deeply involved in critical care nephrology. First, there was consensus that 4% absolute difference in the primary endpoint of all-cause mortality at 90 days could be considered as a MCID. In the main STARRT-AKI analysis, we reported a relative risk of 1.00 (95% CI 0.93–1.09) [3], that is, an absolute difference of 0, with the data being compatible under the null hypothesis to values in the range of a 7% reduction or 9% increase in 90-day all-cause mortality. Therefore, the main analysis was not able to theoretically rule out what could be considered a MCID, as defined by consensus for this analysis, since the 4% absolute reduction was within range of the reported treatment effect size under the frequentist paradigm. The findings of this Bayesian reanalysis can virtually eliminate the possibility that a 4% absolute reduction in the primary endpoint was compatible with the trial data, regardless of the variation in priors used to inform the analysis. Likewise, we were able to conclude with high probability that the accelerated strategy conferred greater KRT dependence, rehospitalization, and fewer KRT-free days when compared to a standard strategy for KRT initiation.

There are limitations to our analysis that warrant consideration. First, this secondary analysis was post hoc; however, we developed an a priori analytic plan prior to data analysis. Second, we recognize that priors used in Bayesian analysis are subjective. To address this, we used a range of priors, including those derived from prior trial data and consensus. Third, we did not impute for missing data. Fourth, we did not adjust for multiplicity of testing, though the concern for type I error may be reduced with Bayesian analysis compared with a frequentist analysis, and our findings were coherent with the main STARRT-AKI trial [3, 11]. Fifth, we used margins for equivalence and for defining large effect sizes that may be questionable; however, we also present results based on consensus definition of MCID, which corroborates with consistent interpretation.

## Conclusions

This Bayesian reanalysis of the STARRT-AKI trial showed that there is a very low probability that an accelerated KRT strategy will lead to a clinically important improvement in 90-day all-cause mortality. In addition, patients who were allocated to the accelerated strategy probably had fewer KRT-free days, and a higher probability of 90-day KRT dependence and rehospitalization. Collectively, these findings do not support adoption of an early or accelerated strategy for KRT initiation.

## Supplementary Information

**Additional file 1.** Analysis overview for the key secondary outcomes.** eTable 1**. Full model report according to theoretical priors.** eFigure 1**. Distribution of days alive and KRT-free according to allocated strategy.** eFigure 2**. Distribution of days alive and free of hospitalization according to allocated strategy. Consensus definitions for equivalence, minimal clinically important difference (MCID), and large effects. STARRT-AKI Investigators.

## Data Availability

The STARRT-AKI has a data sharing policy available at: https://www.ualberta.ca/critical-care/research/current-research/starrtaki/documents.html

## Abbreviations

AKI: Acute kidney injury
CrI: Credible interval
GFR: Glomerular filtration rate
HDI: High-density interval
ICU: Intensive care unit
IPDMA: Individual patient data meta-analysis
KRT: Kidney-replacement therapy
MCID: Minimal clinically important difference
ROPE: Range of practical equivalence
