# Supplementary material for: A Bayesian reanalysis of the Standard versus Accelerated Initiation of Renal-Replacement Therapy in Acute Kidney Injury (STARRT-AKI) trial
Source: Crit Care. 2022 Aug 25;26:255. doi: 10.1186/s13054-022-04120-y (PMC9404618; doi:10.1186/s13054-022-04120-y)
Supplement: Supplementary file 1 — Additional file 1. Analysis overview for the key secondary outcomes. eTable 1. Full model report according to theoretical priors. eFigure 1. Distribution of days alive and KRT-free according to allocated strategy. eFigure 2. Distribution of days alive and free of hospitalization according to allocated strategy. Consensus definitions for equivalence, minimal clinically important difference (MCID), and large effects. STARRT-AKI Investigators. [file 13054_2022_4120_MOESM1_ESM.docx]

Electronic Supplementary Material

A Bayesian Reanalysis of the Standard versus Accelerated Initiation of Renal-Replacement Therapy in Acute Kidney Injury (STARRT-AKI) Trial

**Analysis overview for Primary Endpoint**

All analyses were run under R 4.2.0 using {brms} with {cmdstanr} as backend. Contrasts and sampling for models were made using {emmeans}. We used {dplyr} for data-wrangling and {ggplot2} for plotting.

The primary endpoint was assessed using a hierarchical Bayesian model that followed the following syntax using brms:

brm(death ~ sepsis + surgical + ckd_admission + intervention + (1|site), data = db, family = "bernoulli", iter = 4000, chains = 4, cores = 4, seed = 123, prior = prior, backend = "cmdstanr").

That is, 4 chains with 4,000 iterations were run for the primary endpoint analysis. All priors were assumed to follow a normal distribution. The priors used were: 1. Theoretical priors: These were based on reference [7]. These priors included a neutral moderately skeptical prior, an optimistic moderately skeptical prior, and a pessimistic moderately skeptical prior. The neutral prior followed $N\left( 0,0.355 \right)$, which is centered at zero (absence of effect). The optimistic and pessimistic priors were mirrored around the effect size that the STARRT-AKI trial was designed to detect: A 6% absolute risk reduction in 90-day mortality from 40 to 34%, representing OR = 0.77 [log[OR]=-0.257]). Standard deviation for each prior was set so that the optimistic prior still had a probability mass of benefit close to 0.15, and the pessimistic prior had a probability mass pointing towards benefit of 0.15. This resulted in:

1. $N\left( -0.257,0.249 \right)$ for the optimistic prior.
2. $N\left( 0.257,0.249 \right)$ for the pessimistic priors

Data derived priors were obtained from their respective studies. In brief, we collected the absolute number of events in intervention and control group in the STARRT-AKI pilot trial, the AKIKI trial, the ELAIN trial and the IPDMA. We ran a Bayesian logistic regression on toy data based on each trial numbers with flat priors and used the resulting parameters of the effect size of the intervention as priors for the main analyses. The final priors were:

1. STARRT-AKI Pilot: $N\left( -0.046,0.428 \right)$
2. AKIKI prior: $N\left( -0.066,0.163 \right)$
3. ELAIN: $N\left( -0.623,0.262 \right)$
4. Metanalysis: $N\left( -0.036,0.010 \right)$

Priors for other predictors were set as $N\left( 0,1 \right)$ Results were obtaining using the emmeans package. Pipes from magrittr were used through the code. The following syntax was used to obtain marginal absolute differences:

model %>% emmeans(~ intervention, epred = TRUE, re_formula = NULL) %>% contrast(method = "revpairwise")

Marginal odds ratio was computed by obtaining predicted probabilities for each level of intervention (accelerated or control):

m1 %>% emmeans(~ intervention, epred = TRUE, re_formula = NULL) %>% gather_emmeans_draws() %>% select(-.chain,-.iteration) %>% pivot_wider(names_from = "intervention",values_from = ".value",id_cols = .draw)

From the probabilities, odds ratio were obtaining using the standard formula $\left( P_{accelerated}/\left( 1-P_{accelerated} \right) \right)/\left( P_{control}/\left( 1-P_{control} \right) \right)$. This resulted in a posterior marginal odds ratio distribution, which was summarized using median and standard deviation.

**Analysis Overview for Key Secondary Endpoints**

Days alive and free of KRT was modeled following a zero-one inflated beta regression model. We initially defined the percentage number of days free of KRT the patient had up to 90-days. The model syntax was:

brm(bf(I(days_free_of_krt/90) ~ sepsis + surgical+ ckd_admission + intervention + (1|site), phi ~ sepsis + surgical+ ckd_admission + intervention + (1|site), zoi ~ sepsis + surgical+ ckd_admission + intervention + (1|site), coi ~ sepsis + surgical+ ckd_admission + intervention + (1|site)),...)

Where first component estimated mean, phi estimated variance, zoi estimates the zero-one inflation and coi the conditional one inflation. All were adjusted for main covariates. Results represented the percentage of free-days until 90-days, which were back converted to days by multiplying by 90.

Days alive and free of hospitalization was modeled using zero inflated beta regression model, since we expected no true 1 values (no patient would have 90 days alive and free of hospital since they were randomized in the ICU). Model syntax was to previous model, but zoi and coi were replaced by zi, which is the zero inflation:

brm(bf(I(days_alive_outside_hospital/90) ~ sepsis + surgical+ ckd_admission + intervention + (1|site), phi ~sepsis + surgical+ ckd_admission + intervention + (1|site), zi ~ sepsis + surgical+ ckd_admission + intervention + (1|site)), family = zero_inflated_beta(), ...)

Both models were run with 4 chains and 4,000 iterations with cmdstanr as back-end. We used only neutral priors for the intervention in this analysis, defined as:

nprior <- c(prior(normal(0,0.355), class = b, coef = interventionAccelerated), prior(normal(0,1), class = b))

This model syntax means that for the neutral prior was used for binary component of the models; the remaining priors were uninformative.

**Supplementary Data**

**eTable 1** – Full model report according to theoretical priors.

| Parameter | Median | CrI low | CrI high | PD | % ROPE | Prior |
| --- | --- | --- | --- | --- | --- | --- |
| Intercept | 0.70 | 0.57 | 0.85 | 1.00 | 0.01 | Neutral |
| Sepsis (Yes) | 1.27 | 1.08 | 1.49 | 1.00 | 0.23 | Neutral |
| Surgical (Yes) | 0.66 | 0.55 | 0.78 | 1.00 | 0.00 | Neutral |
| CKD (Yes) | 1.11 | 0.95 | 1.30 | 0.89 | 0.86 | Neutral |
| Accelerated RRT (Yes) | 1.00 | 0.87 | 1.17 | 0.53 | 1.00 | Neutral |
| Intercept | 0.70 | 0.58 | 0.86 | 1.00 | 0.02 | Optimistic |
| Sepsis (Yes) | 1.27 | 1.08 | 1.48 | 1.00 | 0.23 | Optimistic |
| Surgical (Yes) | 0.66 | 0.55 | 0.78 | 1.00 | 0.00 | Optimistic |
| CKD (Yes) | 1.11 | 0.96 | 1.29 | 0.91 | 0.86 | Optimistic |
| Accelerated RRT (Yes) | 0.98 | 0.84 | 1.14 | 0.59 | 1.00 | Optimistic |
| Intercept | 0.69 | 0.57 | 0.84 | 1.00 | 0.01 | Pessimistic |
| Sepsis (Yes) | 1.27 | 1.07 | 1.49 | 1.00 | 0.25 | Pessimistic |
| Surgical (Yes) | 0.66 | 0.55 | 0.78 | 1.00 | 0.00 | Pessimistic |
| CKD (Yes) | 1.10 | 0.94 | 1.30 | 0.90 | 0.87 | Pessimistic |
| Accelerated RRT (Yes) | 1.03 | 0.89 | 1.18 | 0.64 | 1.00 | Pessimistic |

####

#### **eFigure 1** - Distribution of Days alive and KRT-free according to allocated strategy.


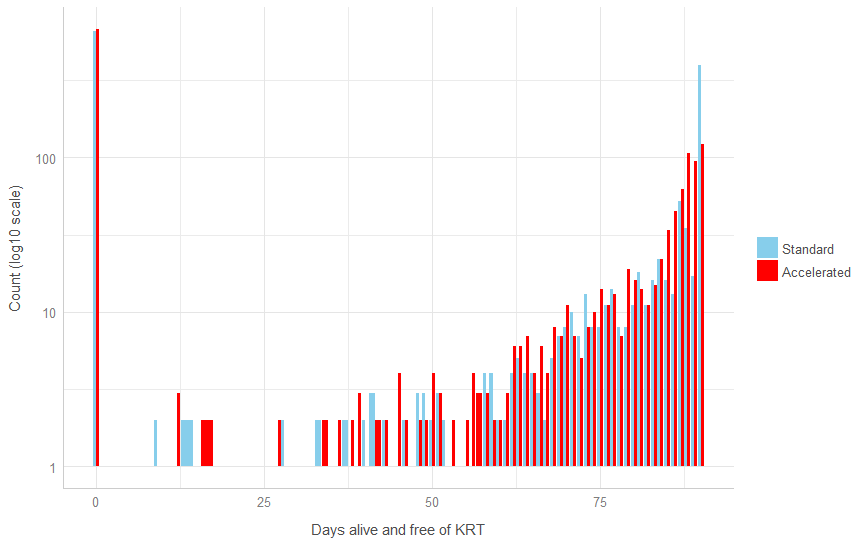


**eFigure 2** – Distribution of days alive and free of hospitalization according to allocated strategy.


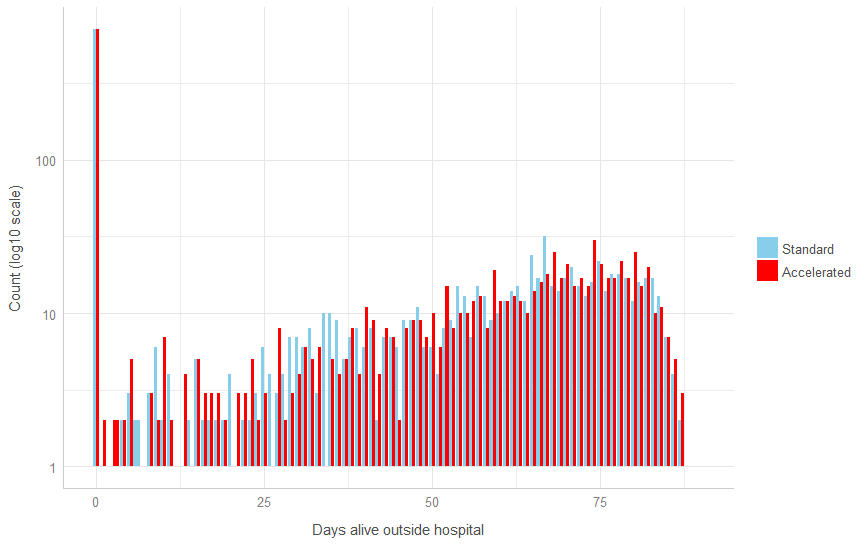


**Consensus Definitions for Equivalence, Minimal Clinically Important Difference (MCID), and Large Effects**

Defining margins for equivalence, MCID and large effects can always be subject to criticisms. Kruschke [1,2] proposed that a standardized mean difference of 0.1 could be considered as a guidance for equivalence in lack of better information. We converted this difference to log(OR) scale which resulted in a margin of equivalence for log odds of |0.18|. Region of practical equivalence (ROPE) was defined as the probability mass of the posterior that fell inside the margins of -0.18 to +0.18, in log(OR) scale.

Defining MCID required additional information and obtaining expert consensus or at least information from individuals involved in clinical trials is advisable. To obtain estimates for MCID, two questions were e-mailed to the international Steering Committee members by STARRT-AKI trial co-chair (Sean Bagshaw). Members were asked to rank their three top choices. Of the 23 steering committee members, 21 returned their answers. We defined *a priori* that the MCID would be defined using the following decisions rules: 1) If the most frequent option elected as first option was also the most frequently option that received any vote, that option would represent MCID by consensus. 2) If the most frequently voted first choice was not the option that received more overall votes, we would define that no clear consensus occurred and would display both options.

The questions were:

1. Primary endpoint (90-day all-cause mortality) - as you know, we assumed the baseline mortality would be 40% for our sample size estimation, so from this reference, what do you believe would be the minimal clinically important difference (MCID) in mortality between the accelerated and standard strategies in the trial? Please provide your top 3 choices (e.g., in terms of preference, my first choice for MCID in 90-day mortality would be XX%, my second XX% and my third XX%)
2. 1% absolute
3. 2% absolute
4. 4% absolute
5. 6% absolute
6. 8% absolute
7. Secondary endpoint (alive and dialysis-free through 90-days) - for this secondary endpoint, what do you believe would be the MCID in the number of days alive and dialysis-free at 90-days would be between the accelerated and standard strategies in the trial? Again, please provide your top 3 choices (e.g., in terms of preference, my first choice for MCID in days alive and dialysis-free would be XX days, my second XX days and my third XX days)
8. 1 day
9. 2 days
10. 3 days
11. 4 days
12. At least 5 days

Distributions of votes for the first question (mortality) are shown below according to all votes and stratified according to preference order:


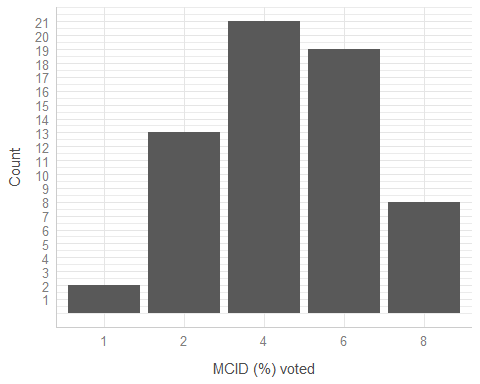

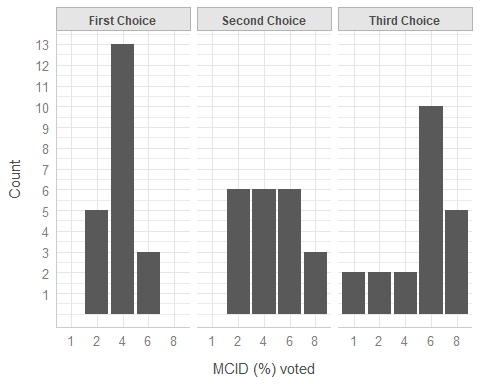


4% was the most common first choice and the option that received more votes in total and was therefore chosen as MCID for the primary endpoint analysis. Assuming a baseline event rate of 40%, this corresponded to an OR equal or below to 0.84. It is important to highlight that an OR of 0.84 translates to an log(OR) of -0.175, which is very similar to the 0.18 margin used as a guide, as suggested by Kruschke. For the “large” effect size for the primary endpoint, we considered that any effect size, in log scale, of 1.5 the effect size defined as equivalent would be considered as “large”. This definition is arbitrary, but it should be highlighted that we present several other posterior summaries in the results to facilitate interpretation.

Distributions of votes for the second question (free-days) are shown below according to all votes and stratified according to preference order:


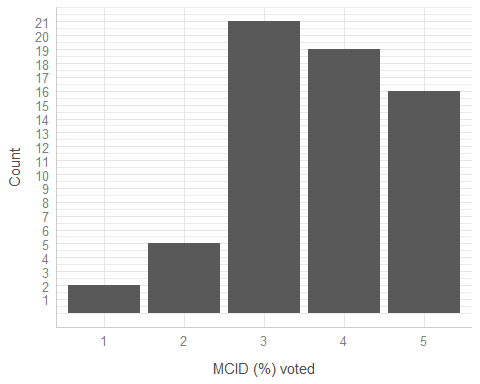

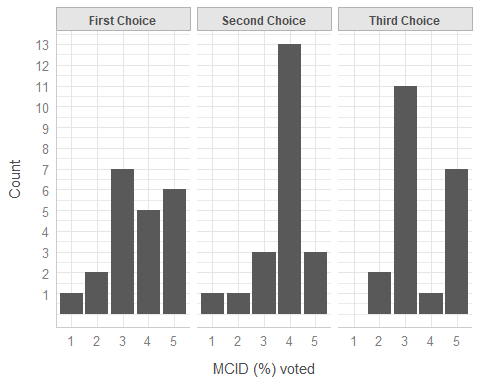


A difference of 3 days was considered as minimal clinically significant, using similar criteria as for mortality (more frequent first option and more frequently any option).

**References:**

1. Kelter R. Analysis of Bayesian posterior significance and effect size indices for the two-sample t-test to support reproducible medical research. BMC Med Res Methodol. 2020 Apr 22;20(1):88. doi: 10.1186/s12874-020-00968-2. PMID: 32321438; PMCID: PMC7178740.

2. Kruschke JK. Doing Bayesian Data Analysis: A Tutorial with R, JAGS, and Stan, Second Edition. Oxford: Academic Press; 2015, pp. 1–759. https://doi.org/10.1016/B978-0-12-405888-0.09999-2. <http://arxiv.org/abs/arXiv:1011.1669v3>.

**STARRT-AKI Investigators:**

| **Institution** | **Name for Publication** |
| --- | --- |
| **Steering Committee** | Sean M Bagshaw (Co-Chair); Ron Wald (Co-Chair); Neill K.J. Adhikari; Rinaldo Bellomo; Didier Dreyfuss; Bin Du; Martin P. Gallagher; Stéphane Gaudry; François Lamontagne; Michael Joannidis; Kathleen D. Liu; Daniel F. McAuley; Shay P. McGuinness; Alistair D. Nichol; Marlies Ostermann; Paul M. Palevsky; Haibo Qiu; Ville Pettilä; Antoine G. Schneider; Orla M. Smith; Suvi Vaara; Matthew Weir |
| **AUSTRALIA** |  |
| Austin Health | Rinaldo Bellomo; Glenn M. Eastwood, Leah Peck; Helen Young |
| Princess Alexandra Hospital | Peter Kruger; Gordon Laurie; Emma Saylor; Jason Meyer; Ellen Venz; Krista Wetzig |
| Western Health | Craig French; Forbes McGain; John Mulder; Gerard Fennessy; Sathyajith Koottayi; Samantha Bates; Miriam Towns; Rebecca Morgan; Anna Tippett |
| The Alfred Hospital | Andrew Udy; Chris Mason; Elisa Licari; Dashiell Gantner; Jason McClure; Alistair Nichol; Phoebe McCracken; Jasmin  Board; Emma Martin; Shirley Vallance; Meredith Young; Chelsey Vladic; Steve McGloughlin |
| Royal Prince Alfred Hospital | David Gattas; Heidi Buhr; Jennifer Coles; Debra Hutch; James Wun |
| Nepean Hospital | Louise Cole; Christina Whitehead; Julie Lowrey; Kristy  Masters; Rebecca Gresham |
| Sunshine Coast University Hospital | Victoria Campbell; David Gutierrez; Jane Brailsford; Loretta Forbes; Lauren Murray; Teena Maguire |
| Barwon Health | Martina NiChonghaile; Neil Orford; Allison Bone; Tania Elderkin; Tania Salerno |
| Bendigo Health | Tim Chimunda; Jason Fletcher; Emma Broadfield; Sanjay Porwal; Cameron Knott; Catherine Boschert; Julie Smith |
| Ballarat Health | Angus Richardson; Dianne Hill |
| Eastern Health | Graeme Duke; Peter Oziemski; Santiago Cegarra; Peter Chan; Deborah Welsh; Stephanie Hunter; Owen Roodenburg; John Dyett; Nicos Kokotsis; Max Moser; Yang Yang; Laven Padayachee; Joseph Vetro; Himangsu Gangopadhyay; Melissa Kaufman |
| The Northern Hospital | Angaj Ghosh; Simone Said |
| Flinders Medical Centre | Alpesh Patel; Shailesh Bihari; Elisha Matheson; Xia Jin; Tapaswi Shrestha; Kate Schwartz |
| Concord Repatriation General Hospital | Martin P. Gallagher; Rosalba Cross; Winston Cheung; Helen Wong; Mark Kol; Asim Shah; Amanda Y. Wang |
| Prince of Wales Clinical School, University of New South Wales | Zoltan Endre |
| Royal North Shore Hospital | Celia Bradford; Pierre Janin; Simon Finfer; Naomi Diel; Jonathan Gatward; Naomi Hammond; Anthony Delaney; Frances Bass; Elizabeth Yarad |
| St. Vincent's Hospital | Hergen Buscher; Claire Reynolds; Nerilee Baker |
| **AUSTRIA** |  |
| Department of Internal Medicine, Medical University Innsbruck | Michael Joannidis; Romuald Bellmann; Andreas Peer; Julia Hasslacher; Paul Koglberger; Sebastian Klein; Klemens Zotter; Anna Brandtner; Armin Finkenstedt; Adelheid Ditlbacher; Frank Hartig |
| Department of General and Surgical Critical Care Medicine, Medical University Innsbruck | Dietmar Fries; Mirjam Bachler; Bettina Schenk; Martin Wagner |
| Department of Internal Medicine, Medical University of Graz | Philipp Eller |
| Medical University of Vienna | Thomas Staudinger; Esther Tiller; Peter Schellongowski; Andja Bojic |
| **BELGIUM** |  |
| Ghent University Hospital | Eric A. Hoste; Stephanie Bracke; Luc De Crop; Daisy Vermeiren |
| **BRAZIL** |  |
| Hospital de Clínicas de Porto Alegre | Fernando Thome; Bianca Chiella; Lucia Fendt; Veronica Antunes |
| **CANADA** |  |
| Centre de recherche de l'Hôpital Maisonneuve-Rosemont | Jean-Philippe Lafrance |
| Centre Hospitalier Universitaire de Sherbrooke | François Lamontagne; Frédérick D'Aragon; Charles St-Arnaud; Michael Mayette; Élaine Carbonnaeu; Joannie Marchand; Marie-Hélène Masse; Marilène Ladouceur |
| CHU de Québec-Université Laval Research Center | Alexis F. Turgeon; François Lauzier; David Bellemare; Charles Langis Francoeur; Guillaume LeBlanc; Gabrielle Guilbault; Stéphanie Grenier; Eve Cloutier; Annick Boivin; Charles Delisle-Thibault; Panagiota Giannakouros; Olivier Costerousse |
| Centre hospitalier de l’Université de Montréal | Jean-François Cailhier; François-Martin Carrier; Ali Ghamraoui; Martine Lebrasseur; Fatna Benettaib; Maya Salamé; Dounia Boumahni |
| Centre intégré universitaire de santé et de services sociaux de la Mauricie-et-du-Centre-du- Québec | Ying Tung Sia; Jean-François Naud; Isabelle Roy |
| Foothills Medical Centre | Henry T. Stelfox; Stacey Ruddell; Braden J. Manns |
| Grey Nuns Community Hospital | Shelley Duggan; Dominic Carney; Jennifer Barchard |
| Hamilton Health Sciences | Richard P. Whitlock; Emilie Belley-Cote; Nevena Savija; Alexandra Sabev; Troy Campbell; Thais Creary; Kelson Devereaux; Shira Brodutch |
| Health Sciences Centre, University of Manitoba | Claudio Rigatto; Bojan Paunovic; Owen Mooney; Anna Glybina; Oksana Harasemiw; Michelle Di Nella |
| Health Sciences North | John Harmon; Navdeep Mehta; Louis Lakatos; Nicole Haslam |
| Institut Universitaire de Cardiologie et de Pneumologie de Québec | Francois Lellouche; Mathieu Simon; Ying Tung; Patricia Lizotte; Pierre-Alexandre Bourchard |
| Juravinski Hospital | Bram Rochwerg; Tim Karachi; Tina Millen |
| Kingston Health Sciences Centre | John Muscedere; David Maslove; J. Gordon Boyd; Stephanie Sibley; John Drover; Miranda Hunt; Ilinca Georgescu |
| Lakeridge Health | Randy Wax; Ilan Lenga; Kavita Sridhar; Andrew Steele; Kelly Fusco; Taneera Ghate; Michael Tolibas; Holly Robinson |
| London Health Sciences Centre, University Hospital | Matthew A. Weir; Ravi Taneja |
| London Health Sciences Centre, Victoria Hospital | Ian M. Ball; Amit Garg; Eileen Campbell; Athena Ovsenek |
| Mazankowski Alberta Heart Institute | Sean M. Bagshaw, Sean van Diepen, Nadia Baig |
| McGill University Health Centre | Sheldon Magder; Han Yao; Ahsan Alam; Josie Campisi |
| Misericordia Community Hospital | Erika MacIntyre; Ella Rokosh; Kimberly Scherr |
| Mount Sinai Hospital | Stephen Lapinsky; Sangeeta Mehta; Sumesh Shah |
| Peter Lougheed Centre | Daniel J. Niven; Henry T. Stelfox; Stacey Ruddell |
| Red Deer Regional Hospital | Michael Russell; Kym Jim; Gillian Brown; Kerry Oxtoby; Adam Hall; Luc Benoit; Colleen Sokolowski |
| Regina Qu'Appelle Health Authority | Bhanu Prasad; Jag Rao; Shelley Giebel |
| Royal Alexandra Hospital | Demetrios J. Kutsogiannis; Patricia Thompson; Tayne Thompson |
| St. Joseph's Health Centre | Robert Cirone; Kanthi Kavikondala |
| St. Joseph's Healthcare | Mark Soth; France Clarke; Alyson Takaoka |
| St. Michael's Hospital | Ron Wald; David Mazer; Karen Burns; Jan Friedrich; David Klein; Gyan Sandhu; Marlene Santos; Imrana Khalid; Jennifer Hodder |
| St. Paul's Hospital | Peter Dodek; Najib Ayas; Victoria Alcuaz |
| Sturgeon Community Hospital | Gabriel Suen; Oleksa Rewa; Gurmeet Singh; Sean Norris; Neil Gibson; Castro Arias; Aysha Shami; Celine Pelletier |
| Sunnybrook Health Sciences Centre | Neill K.J. Adhikari; Alireza Zahirieh; Andre Amaral; Nicole Marinoff; Navjot Kaur; Adic Perez; Jane Wang |
| Surrey Memorial Hospital | Gregory Haljan; Christopher Condin |
| The Ottawa Hospital | Lauralyn McIntyre; Brigette Gomes; Rebecca Porteous; Irene Watpool; Swapnil Hiremath; Edward Clark |
| Toronto General Hospital | Margaret S. Herridge; Felicity Backhouse |
| Toronto Western Hospital | M. Elizabeth Wilcox; Karolina Walczak |
| Trillium Health Partners | Vincent Ki; Asheer Sharman; Martin Romano |
| University of Alberta Hospital | Sean M. Bagshaw; R.T. Noel Gibney; Adam S. Romanovsky; Oleksa Rewa; Lorena McCoshen; Nadia Baig |
| Vancouver Island Health Authority | Gordon Wood; Daniel Ovakim; Fiona Auld; Gayle Carney |
| **CHINA** |  |
| Beijing Friendship Hospital, Capital Medical University | Meili Duan; Xiaojun Ji; Dongchen Guo; Zhili Qi; Jin Lin; Meng Zhang; Lei Dong; Jingfeng Liu; Pei Liu; Deyuan Zhi; Guoqiang  Bai; Yu Qiu; Ziqi Yang; Jing Bai; Zhuang Liu; Haizhou Zhuang; Haiman Wang; Jian Li; Mengya Zhao; Xiao Zhou |
| Guizhou Provincial People's Hospital | Xianqing Shi; Baning Ye; Manli Liu; Jing Wu; Yongjian Fu; Dali Long; Yu Pan; Jinlong Wang; Huaxian Mei; Songsong Zhang; Mingxiang Wen; Enyu Yang; Sijie Mu; Jianquan Li; Tingting Hu |
| Henan Provincial People's Hospital | Bingyu Qin; Min Li; Cunzhen Wang; Xin Dong; Kaiwu Wang; Haibo Wang; Jianxu Yang |
| Peking Union Medical College  Hospital | Bin Du; Chuanyao Wang |
| Peking University First Hospital | Dongxin Wang; Nan Li |
| Renmin Hospital of Wuhan University | Zhui Yu; Song Xu; Lan Yao; Guo Hou; Zhou Liu; Liping Lu; Yingtao Lian |
| Shandong Provincial Hospital | Chunting Wang; Jichen Zhang; Ruiqi Ding; Guoqing Qi; Qizhi Wang; Peng Wang; Zhaoli Meng; Man Chen; Xiaobo Hu |
| The First Affiliated Hospital of Bengbu Medical College | Xiandi He; Shibing Zhao; Lele Hang; Rui Li; Suhui Qin; Kun Lu; Shijuan Dun; Cheng Liu; Qi Zhou; Zhenzhen Chen; Jing Mei |
| The First Affiliated Hospital of Xiamen University | Minwei Zhang; Hao Xu; Jincan Lin |
| The First Affiliated Hospital of Xi'An Jiaotong University | Qindong Shi; Lijuan Fu; Qinjing Zeng; Hongye Ma; Jinqi Yan; Lan Gao; Hongjuan Liu; Lei Zhang; Hao Li; Xiaona He; Jingqun Fan; Litao Guo; Yu Liu; Xue Wang; Jingjing Sun |
| The First Hospital of Jilin University | Zhongmin Liu; Juan Yang; Lili Ding; Lulu Sheng; Xingang Liu |
| Wuxi People's Hospital | Jie Yan; Quihui Wang; Yifeng Wang; Dan Zhao |
| Xiangya Hospital Central South University | Shuangping Zhao; Chenghuan Hu; Jing Li; Fuxing Deng |
| Zhongda Hospital Southeast University | Haibo Qui; Yi Yang; Min Mo; Chun Pan; Changde Wu; Yingzi Huang; Lili Huang; Airan Liu |
| **FINLAND** |  |
| Helsinki University Hospital | Ville Pettilä; Suvi T. Vaara; Anna-Maija Korhonen; Sanna Törnblom; Sari Sutinen; Leena Pettilä; Jonna Heinonen; Eliria Lappi; Taria Suhonen |
| Tampere University Hospital | Sari Karlsson; Sanna Hoppu; Ville Jalkanen; Anne Kuitunen; Markus Levoranta; Jaakko Långsjö; Sanna Ristimäki; Kaisa Malila; Anna Wootten; Simo Varila |
| Turku University Hospital | Mikko J Järvisalo; Outi Inkinen; Satu Kentala; Keijo Leivo; Paivi Haltia |
| **FRANCE** |  |
| Hôpital Louis Mourier | Didier Dreyfuss; Jean-Damien Ricard; Jonathan Messika; Abirami Tiagarajah; Malo Emery; Aline Dechanet; Coralie Gernez; Damien Roux |
| Centre Hospitalier Départemental La Roche-Sur- Yon | Laurent Martin-Lefevre; Maud Fiancette; Isabelle Vinatier; Jean Claude Lacherade; Gwenhaël Colin; Christine Lebert; Marie-Ange Azais; Aihem Yehia; Caroline Pouplet; Matthieu Henry- Lagarrigue; Amélie Seguin; Laura Crosby |
| Medical Intensive Care Unit, Amiens University Hospital | Julien Maizel; Dimitri Titeca-Beauport |
| Hôpital Pitie-Salpetriere | Alain Combes; Ania Nieszkowska; Paul Masi; Alexandre Demoule; Julien Mayaux; Martin Dres; Elise Morawiec; Maxens Decalvele; Suela Demiri; Morgane Faure; Clémence Marios; Maxime Mallet; Marie Amélie Ordon; Laura Morizot; Marie Cantien; François Pousset |
| Hôpital Avicenne/Hôpital Jean Verdier | Stéphane Gaudry; Florent Poirson; Yves Cohen |
| Hospices Civils de Lyon, Hôpital Edouard Herriot, Service de Médecine Intensive – Réanimation | Laurent Argaud; Martin Cour; Laurent Bitker; Marie Simon; Romain Hernu; Thomas Baudry; Sylvie De La Salle |
| CH De Bourg-en-Bresse – Fleyriat | Adrien Robine; Nicholas Sedillot; Xavier Tchenio; Camille Bouisse; Sylvie Roux |
| CHRU de Nîmes | Saber Davide Barbar; Rémi Trusson |
| Rouen University Hospital | Fabienne Tamion; Steven Grangé; Dorothée Carpentier |
| CH Sud Francilien | Guillaume Chevrel; Luis Ensenyat-Martin; Sophie Marque |
| CHU Dijon | Jean-Pierre Quenot; Pascal Andreu; Auguste Dargent; Audrey Large |
| CH Le Mans - Réanimation Medico - Chirurgicale | Nicolas Chudeau; Mickael Landais; Benoit Derrien; Jean Christophe Callahan; Christophe Guitton; Charlène Le Moal; Alain Robert |
| CHU Nantes/Service d'Anesthésie - Réanimation chirurgicale HD PTMC | Karim Asehnoune; Raphaël Cinotti; Nicolas Grillot; Dominique Demeure |
| Germon et Gauthier Hospital – Béthune | Christophe Vinsonneau; Imen Rahmani; Mehdi Marzouk; Thibault Dekeyser; Caroline Sejourne; Mélanie Verlay; Fabienne Thevenin; Lucie Delecolle |
| Centre Hospitalier Lens | Didier Thevenin |
| Clermont Ferrand | Bertrand Souweine; Elisabeth Coupez; Mireille Adda |
| CH de Dieppe | Jean-Pierre Eraldi; Antoine Marchalot |
| Hôpital Henri Mondor | Nicolas De Prost; Armand Mekontso Dessap; Keyvan Razazi |
| Hôpital Civil | Ferhat Meziani; Julie Boisrame-Helms; Raphael Clere-Jehl; Xavier Delabranche; Christine Kummerlen; Hamid Merdji; Alexandra Monnier; Yannick Rabouel; Hassene Rahmani; Hayat Allam; Samir Chenaf; Vincenta Franja |
| CHU de Pointe à Pitre | Bertrand Pons; Michel Carles; Frédéric Martino; Régine Richard |
| André Mignot | Benjamin Zuber; Guillaume Lacave |
| CHU de Nantes | Karim Lakhal; Bertrand Rozec; Hoa Dang Van |
| Centre de Beaumont sur Oise | Éric Boulet |
| Centre Hospitalier René Dubos Pontoise | Fouad Fadel; Cedric Cleophax; Nicolas Dufour; Caroline Grant; Marie Thuong |
| Hotel Dieu – Service de Médicale | Jean Reignier; Emmanuel Canet; Laurent Nicolet |
| CHR Orleans | Thierry Boulain; Mai-Anh Nay; Dalila Benzekri; François Barbier; Anne Bretagnol; Toufik Kamel; Armelle Mathonnet; Grégoire Muller; Marie Skarzynski; Julie Rossi; Amandine Pradet; Sandra Dos Santos; Aurore Guery; Lucie Muller; Luis Felix |
| CH Lyon Sud – Pierre Benite | Julien Bohé; Guillaume Thiéry |
| Universite de Paris, Hopital Europeen Georges Pompidou | Nadia Aissaoui; Damien Vimpere; Morgane Commeureuc; Jean-Luc Diehl; Emmanuel Guerot |
| **GERMANY** |  |
| Klinikum Coburg | Orfeas Liangos; Monika Wittig |
| University Hospital Münster | Alexander Zarbock; Mira Küllmar; Thomas van Waegeningh; Nadine Rosenow |
| **IRELAND** |  |
| St. Vincent's University Hospital | Alistair D. Nichol; Kathy Brickell; Peter Doran; Patrick T. Murray |
| **ITALY** |  |
| IRCCS San Raffaele Scientific Institute | Giovanni Landoni; Rosalba Lembo; Alberto Zangrillo; Giacomo Monti; Margherita Tozzi; Matteo Marzaroli; Gaetano Lombardi |
| San Carlo Hospital | Gianluca Paternoster; Michelangelo Vitiello |
| **NEW ZEALAND** |  |
| Cardiovascular Surgical Intensive Care Unit, Auckland Hospital | Shay McGuinness; Rachael Parke; Magdalena Butler; Eileen Gilder; Keri-Anne Cowdrey; Samantha Wallace; Jane Hallion; Melissa Woolett; Philippa Neal; Karina Duffy;  Stephanie Long |
| Department of Critical Care Medicine, Auckland Hospital | Colin McArthur; Catherine Simmonds; Yan Chen; Rachael McConnochie; Lynette Newby |
| Christchurch Hospital | David Knight; Seton Henderson; Jan Mehrtens; Stacey Morgan; Anna Morris; Kymbalee Vander Hayden; Tara Burke |
| Hawke's Bay Hospital | Matthew Bailey; Ross Freebairn; Lesley Chadwick; Penelope Park; Christine Rolls; Liz Thomas |
| Rotorua Hospital | Ulrike Buehner; Erin Williams |
| Taranaki Hospital | Jonathan Albrett; Simon Kirkham; Carolyn Jackson |
| Tauranga Hospital | Troy Browne; Jennifer Goodson; David Jackson; James Houghton; Owen Callender; Vicki Higson; Owen Keet; Clive Dominy |
| Wellington Hospital | Paul Young; Anna Hunt; Harriet Judd; Cassie Lawrence; Shaanti Olatunji; Yvonne Robertson; Charlotte Latimer-Bell; Deborah Hendry; Agnes Mckay-Vucago; Nina Beehre; Eden  Lesona; Leanlove Navarra; Chelsea Robinson |
| Whangarei Hospital | Ryan Jang; Andrea Junge; Bridget Lambert |
| **SWITZERLAND** |  |
| Centre Hospitalier Universitaire Vaudois | Antoine G. Schneider; Michel Thibault; Philippe Eckert; Sébastien Kissling; Erietta Polychronopoulos; Elettra Poli; Marco Altarelli; Madeleine Schnorf; Samia Abed Mallaird |
| Hôpitaux Universitaires de Genève | Claudia Heidegger; Aurelie Perret; Philippe Montillier; Frederic Sangla; Seigenthaller Neils; Aude De Watteville |
| **UNITED KINGDOM** |  |
| Barking, Havering and  Redbridge University Hospitals NHS Trust | Mandeep-Kaur Phull; Aparna George; Nauman Hussain; Tatiana Pogreban |
| Barnsley Hospital NHS Foundation Trust | Steve Lobaz; Alison Daniels; Mishell Cunningham; Deborah Kerr; Alice Nicholson |
| Buckinghamshire Healthcare NHS Trust | Pradeep Shanmugasundaram; Judith Abrams; Katarina Manso; Geraldine Hambrook; Elizabeth McKerrow; Juvy Salva; Stephen Foulkes |
| Cardiff and Vale University Health Board | Matthew Wise; Matt Morgan; Jenny Brooks; Jade Cole; Tracy Michelle Davies; Helen Hill; Emma Thomas |
| Chelsea and Westminster Hospital NHS Foundation Trust | Marcela Vizcaychipi; Behrad Baharlo; Jaime Carungcong; Patricia Costa; Laura Martins |
| East Kent NHS Trust | Ritoo Kapoor; Tracy Hazelton; Angela Moon; Janine  Musselwhite |
| Golden Jubilee National Hospital, NHS Scotland | Ben Shelley; Philip McCall |
| Guy's and St. Thomas’ NHS Foundation Trust | Marlies Ostermann; Gill Arbane; Aneta Bociek; Martina Marotti; Rosario Lim; Sara Campos; Neus Grau Novellas; Armando Cennamo; Andrew Slack; Duncan Wyncoll; Luigi Camporota; Simon Sparkes; Rosalinde Tilley |
| University Hairmyres Hospital, NHS Lanarkshire | Austin Rattray; Gayle Moreland; Jane Duffy; Elizabeth McGonigal |
| King's College Hospital NHS Foundation Trust | Philip Hopkins; Clare Finney; John Smith; Harriet Noble; Hayley Watson; Claire-Louise Harris; Emma Clarey; Eleanor Corcoran |
| Leeds Teaching Hospital NHS Foundation Trust | James Beck; Clare Howcroft; Nora Youngs; Elizabeth Wilby; Bethan Ogg |
| Lincoln County Hospital – United Lincolnshire Hospitals NHS Foundation Trust | Adam Wolverson; Sandra Lee; Susie Butler; Maryanne Okubanjo; Julia Hindle |
| Liverpool University Hospitals NHS Foundation Trust | Ingeborg Welters; Karen Williams; Emily Johnson; Julie Patrick-Heselton; David Shaw; Victoria Waugh |
| Milton Keynes University Hospital NHS Foundation Trust | Richard Stewart; Esther Mwaura; Lynn Wren; Louise Mew; Sara-Beth Sutherland; Jane Adderley |
| University Hospital Monklands, NHS Lanarkshire | Jim Ruddy; Margaret Harkins |
| NHS Grampian | Callum Kaye; Teresa Scott; Wendy Mitchell; Felicity Anderson;  Fiona Willox |
| North Tees and Hartlepool Foundation NHS Trust | Vijay Jagannathan; Michele Clark; Sarah Purv |
| Nottingham University Hospital - Queen's Medical Centre | Andrew Sharman; Megan Meredith; Lucy Ryan; Louise Conner; Cecilia Peters; Dan Harvey |
| Queen Elizabeth Hospital - Lewisham and Greenwich NHS Trust | Ashraf Roshdy; Amy Collins |
| Queen Elizabeth University Hospital | Malcolm Sim; Steven Henderson |
| Royal Bournemouth & Christchurch Hospitals NHS Trust | Nigel Chee; Sally Pitts; Katie Bowman; Maria Dilawershah; Luke Vamplew; Elizabeth Howe |
| Royal Brompton and Harefield NHS Foundation Trust | Paula Rogers; Clara Hernandez; Clara Prendergast; Jane Benton; Alex Rosenberg |
| Royal Surrey County Hospital  NHS Foundation Trust | Lui G. Forni; Alice Grant; Paula Carvelli |
| Sheffield Teaching Hospitals NHS Foundation Trust | Ajay Raithatha; Sarah Bird; Max Richardson; Matthew Needham; Claire Hirst |
| St. George’s University Hospitals NHS Foundation Trust | Jonathan Ball; Susannah Leaver; Luisa Howlett; Carlos Castro Delgado; Sarah Farnell-Ward; Helen Farrah; Geraldine Gray;  Gipsy Joseph; Francesca Robinson |
| St. Helen's and Knowsley Teaching Hospitals NHS Trust | Ascanio Tridente; Clare Harrop; Karen Shuker |
| University Hospital Ayr, NHS Ayrshire & Arran | Derek McLaughlan; Judith Ramsey; Sharon Meehan |
| University Hospital Lewisham, Lewisham and Greenwich NHS Trust | Bernd Oliver Rose; Rosie Reece-Anthony; Babita Gurung |
| University Hospitals Birmingham NHS Foundation Trust | Tony Whitehouse; Catherine Snelson; Tonny Veenith; Andy Johnston; Lauren Cooper; Ron Carrera; Karen Ellis; Emma Fellows; Samanth Harkett; Colin Bergin; Elaine Spruce; Liesl Despy; Stephanie Goundry; Natalie Dooley; Tracy Mason; Amy Clark |
| University Hospitals Coventry and Warwickshire NHS Trust | Gemma Dignam; Geraldine Ward |
| Warwick Hospital, South Warwickshire NHS Trust | Ben Attwood; Penny Parsons; Sophie Mason |
| St. Richard's Hospital, Western  Sussex Hospitals NHS Foundation Trust | Michael Margarson; Jenny Lord; Philip McGlone |
| Worthing Hospital, Western Sussex Hospitals NHS Foundation Trust | Luke E. Hodgson; Indra Chadbourn; Raquel Gomez; Jordi Margalef |
| York Teaching Hospital NHS Foundation Trust | Rinus Pretorius; Alexandra Hamshere; Joseph Carter; Hazel Cahill; Lia Grainger; Kate Howard; Greg Forshaw; Zoe Guy |
| **UNITED STATES** |  |
| Mayo Clinic, Rochester | Kianoush B. Kashani; Robert C. Albright Jr.; Amy Amsbaugh; Anita Stoltenberg; Alexander S. Niven |
| Rhode Island Hospital | Matthew Lynch; AnnMarie O'Mara; Syed Naeem; Sairah Sharif; Joyce McKenney Goulart |
| The Miriam Hospital | Matthew Lynch; AnnMarie O'Mara; Syed Naeem; Sairah Sharif; Joyce McKenney Goulart |
| University of Alabama at Birmingham | Ashita Tolwani; Claretha Lyas; Laura Latta |
| University of Florida | Azra Bihorac; Haleh Hashemighouchani; Philip Efron; Matthew Ruppert; Julie Cupka; Sean Kiley; Joshua Carson; Peggy White; George Omalay; Sherry Brown; Laura Velez; Alina  Marceron |
| University of Kentucky | Javier A. Neyra; Juan Carlos Aycinena; Madona Elias; Victor  M. Ortiz-Soriano; Caroline Hauschild; Robert Dorfman |
